# Editors’ Book Table

**Published:** 1872-03

**Authors:** 


### Editors' Book Table.

[NOTE. — All works reviewed in the columns of the CHICAGO MEDICAL JOURNAL may be found in the extensive stock of W. B. KEEN, COOKE & CO., whose catalogue of Medical Books will be sent to any address upon request.]

BOOKS RECEIVED.

*Animal and Vegetable Parasites of the Human Skin and Hair.* By B. JOY JEFFRIES, A.M., M.D., etc., etc. Boston: Alexander Moore. 1872. $1.00.

A concise, clear and practical exposition of the subject. Several wood cuts are given, which facilitate understanding of the text.

*Plain Talk about Insanity: Its Causes, Forms and Symptoms, and the Treatment of Mental Diseases; with Remarks on Hospitals and Asylums, and the medico-legal aspect of Insanity.* By T. W. FISHER, M.D., late of the Boston Hospital for the Insane. Boston: Alexander Moore. 1872. $1.50.

A book which concentrates, in small bulk, a vast amount of valuable and suggestive thought. We have not read for a long time a book or essay on the subject which we could as confidently recommend to our readers. It is clear in statement, philosophical in its development, and thoroughly *common sense* in its teachings. To buy it is to put your money to excellent use.

PAMPHLETS.

*Small-Pox: The Predisposing Conditions and their Prevention.* By Dr. CARL BOTH. Boston: Alexander Moore. 1872. 25 cents.

This *brochure* has attracted much attention both from the professional and secular press. The author concludes that vaccination is not an unfailing protective measure, and that something more sure and certain in its effects is demanded. Studying the relation of the blood-salts, especially table salt, to the albumen, he finds that they perform the office of keeping it in proper balance. If salt is wanting, albumen will be in excess; if it preponderates, albumen is deficient or in demand. The inconveniences attending these extremes are sufficiently obvious. The *predisposition* to small-pox, for instance, "consists in an undue proportion of albuminous matter to the blood-salts, and that as the result, an otherwise inoffensive nervous irritation becomes sufficient to cause the blood to part with this superfluous albumen, which in this case is thrown into the skin, and constitutes that condition which is commonly called small-pox."

He further maintains "that a person who does not exhibit this superabundance of albuminous matter in the blood is *not* liable to small-pox under any circumstances of exposure or contact with patients suffering from this disorder." Hence, he argues, small-pox always occurs when salt is scarce or absent. It is more likely to occur to spirit drinkers, because alcohol throws out their blood-salts, producing a preponderance of albumen. The Prussian army had no small pox in the late war because they were well provided with *pea sausages*, which contained not only salt but *all* the necessary ingredients the human body requires for health and vigor.

Not only alcohol but sugar is vicious in this connection, because it satisfies the taste which otherwise would demand salt. But as sugar cannot replace salt in its uses in the blood, when persons employ it to the exclusion of salt they are thereby rendered more liable to infection from small-pox.

Unfortunately, it will not do for those who have erred in these respects to rush, incontinently, into the excessive use of salt, for according to Dr. Both, who italicizes the proposition, this may be the immediate occasion of the appearance of the dreaded disease. It is better in these cases to first give organic acids, lemon juice, etc., and then gradually introduce the saline.

In American cities, 75 out of 100 children are fed too little salt. It should be used in puddings before sugar. Incidentally, he claims that tea, coffee, alcoholic stimulants, and even milk, should not be taken with the meals. The close is as follows:

"*Resume.*—Let it be distinctly understood that what I intend to say is, that vaccination has not proved to be an absolutely reliable preventive against small-pox; and, therefore, that better means for protection are needed.

"That it is absolutely necessary, first, to know the nature of a disease before any attempt can be made to prevent it.

"That I have discovered the following facts, and maintain, that small-pox consists in the escape of *superfluous* albuminous substances into the tissues of the periphery of the nervous centres of the body, caused, in the first place, by the want of salt.

"That the *proper* use of salt is the scientific and most certain preventive of small-pox, both in theory and practice, that I have any knowledge of.

"That the use of organic acids is the best means of freeing the blood from abnormal substances, which, for the time being, may substitute the place of salt in the body.

"That alcohol is an agent which eliminates the blood-salts, and, therefore, after its use, the salt thus eliminated must be restored.

"That sugar can take the place of salt in regard to taste, much to the injury of the blood of the person substituting it.

"That in mental labor more salt is brought into requisition, and used up, than in muscular or physical labor; and, therefore, that more must be used or taken into the body in the former case than in the latter.

"That a person who has a properly balanced blood cannot catch, or take, small-pox under any circumstances of exposure.

"This theory, if correct, must hold good in all cases, without a single exception; and if nothing can be found to disprove its correctness, it holds good that the *proper* use of salt in the human economy, will eradicate small-pox at once and forever.

"Therefore the use and office of salt should be more generally known and taught in all our public schools."

